# Deficiency of valencene in mandarin hybrids is associated with a deletion in the promoter region of the valencene synthase gene

**DOI:** 10.1186/s12870-019-1701-6

**Published:** 2019-03-13

**Authors:** Qibin Yu, Ming Huang, Hongge Jia, Yuan Yu, Anne Plotto, Elizabeth A. Baldwin, Jinhe Bai, Nian Wang, Frederick G. Gmitter Jr

**Affiliations:** 1 University of Florida, Institute of Food and Agricultural Sciences, Citrus Research and Education Center, Lake Alfred, FL 33850 USA; 2 USDA-ARS Horticultural Research Laboratory, Fort Pierce, FL 34945 USA

**Keywords:** Cloning, QTL mapping, Sesquiterpene, cDNA sequencing, Transient expression, Citrus

## Abstract

**Background:**

Valencene is a major sesquiterpene in citrus oil and biosynthesized by valencene synthase (Cstps1; EC: 4.2.3.73) from the 15-carbon substrate farnesyl diphosphate. It is abundant in juice of some mandarins (e.g. *Citrus reticulata* Blanco cv. Fortune), however, it is undetectable in others (e.g. *C. reticulata* Blanco cv. Murcott), We have discovered that the Murcott mandarin *Cstps1* gene expression is severely reduced. A previous genetic mapping study using an F1 population of Fortune × Murcott found that the segregation of valencene production in fruit exhibited a Mendelian inheritance ratio of 1:1. There was only one dominant locus associated with valencene content detected on the mandarin genetic map. The goal of this study was to understand the molecular mechanism underlying the valencene deficiency observed in some citrus hybrids.

**Results:**

There was a clear relationship between presence or absence of the valencene synthase gene (*Cstps1*) expression, and presence or absence of valencene among randomly selected mandarin hybrids. Cloning the coding regions of *Cstps1* from Fortune and Murcott mandarin, and aligning with previous reported Valencia orange *Cstps1* sequence, showed that they both exhibited extremely high similarity with the known *Cstps1*. By further cloning and analyzing the promoter region of *Cstps1* from Valencia, Fortune and Murcott, a 12-nucleotide deletion at approximately − 270 bp from the *Cstps1* coding region was only found in Murcott. Three binary vectors, designated as p1380-FortP-GUSin, p1380-MurcP-GUSin and p1380-MurcP(+ 12)-GUSin, were developed for promoter activity analysis. Transient over-expression of Fortune *Cstps1* promoter in sweet orange showed notable GUS activity, but the Murcott *Cstps1* promoter did not. In addition, by re-inserting the 12-nucleotide fragment, the activity of the Murcott *Cstps1* promoter was mostly recovered.

**Conclusion:**

The deficiency of valencene production in some mandarins is probably due to a 12-nucleotide deletion in the promoter region of the *Cstps1*, which could be a crucial switch of *Cstps1* transcription. Our results further enhanced the understanding of valencene biosynthesis in citrus.

**Electronic supplementary material:**

The online version of this article (10.1186/s12870-019-1701-6) contains supplementary material, which is available to authorized users.

## Background

**Fig. 1:**
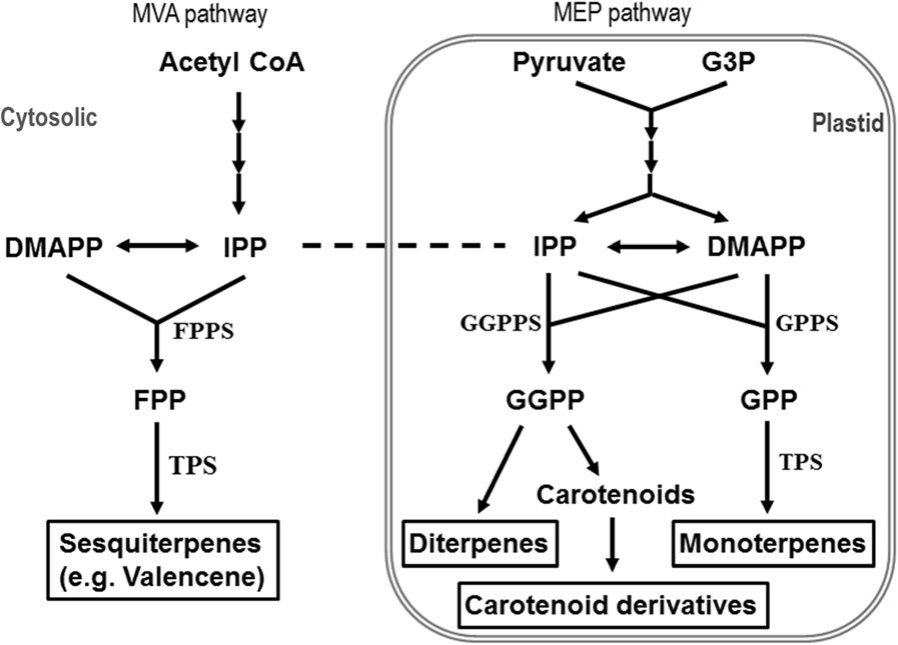
Schematic pathways of terpene biosynthesis. DMAPP, dimethylallyl pyrophosphate; FPP, farnesyl pyrophosphate; FPPS, FPP synthase; G3P, glyceraldehyde-3-phosphate; GGPP, geranylgeranyl pyrophosphate; GGPPS, GGPP synthase; GPP, geranyl pyrophosphate; GPPS, GPP synthase; IPP, isopentenyl pyrophosphate; MEP, methylerythritol phosphate; MVA, mevalonic acid; TPS, terpene synthase

Mandarin hybrids are commercial citrus that produce small, easily peeled, sweet fruit with delicate and pleasant flavor, and a forecasted increased production of 28–30 million ton globally per year. Since high quality citrus fruit can lead to greater economic returns for the industry, improvement in fruit aroma and flavor has become one of the primary goals of fresh citrus fruit breeding programs. Breeding for citrus flavor has been hampered by challenges in screening the large range of metabolic chemicals. Therefore, it is important to understand the complex production and regulatory mechanisms of fruit flavor compounds to lead to efficient breeding strategies. Fruit flavor perception includes sweetness (glucose, sucrose and fructose), sourness (citric and malic acids) and bitterness (flavonoids and limonoids), in addition to aroma compounds [1, 2]. Our previous studies determined 60–70 different volatile compounds for 30 mandarin hybrids in the University of Florida citrus breeding program [3, 4]. Aroma in mandarin fruit is due to complex combinations of several chemical families such as terpenes, hydrocarbons, aldehydes, esters, alcohols, ketones and sulfur compounds. Terpenes play an important role in generating citrus fruit aroma, accounting for 85–95% of volatiles in mandarin fruit, and provide pleasant green, piney and citrus aromas [4]. The diversity of terpenes seems to stem mainly from the specific composition and expression of terpene synthases, the key enzymes in the biosynthetic pathway, and additional modification controlled by downstream enzymes [5]. Despite their diversity, all terpenes originate from isopentenyl diphosphate (IPP) and its allylic isomer dimethylallyl diphosphate (DMAPP). Prenyltransferase condenses DMAPP with two IPP molecules to produce farnesyl pyrophosphate (FPP) or three IPPs to form geranylgeranyl diphosphate (GGPP) (Fig. 1). In the cytosol, the mevalonate (MVA) pathway synthesizes sesquiterpenes, phytosterols and ubiquinone, whereas the methylerythritol 4-phosphate (MEP) pathway produces monoterpenes, gibberellins, abscisic acid, carotenoids and the prenyl moiety of chlorophylls, plastoquinone and tocopherol in plastids [6, 7].

Valencene is a major sesquiterpene in citrus oil, the second most abundant volatile after limonene, which accounts for 90% of orange/tangerine essential oil. Nootkatone, a putative derivative of valencene, has a dominant role in the flavor and aroma of grapefruit. Valencene concentration increases as fruits mature and the flavor quality develops [5, 8, 9]. Elston et al. [8] suggested that valencene may be marker with a statistical correlation to higher quality in orange oil simply because it correlates with maturity, which give citrus juice flavor and fragrance. Both valencene and nootkatone are used for flavoring citrus beverages and are considered among the most valuable terpenes used on a commercial scale. Sharon-Asa et al. [5] reported that *Cstps1* encodes valencene synthase, a sesquiterpene synthase catalyzing the biosynthesis of valencene from the 15-carbon substrate farnesyl diphosphate. Our previous study [10] found that valencene accounted for 9.4% of the total volatile content in fruit of the mandarin hybrid, Temple, whereas no valencene was detected in fruit of the mandarin hybrid, Murcott. In addition, the expression level of the *Cstps1* gene is severely reduced in Murcott [9]. The molecular mechanism for valencene deficiency in Murcott would be of particular interest for verification of the biological role of terpene synthases in the terpenoid pathway. The diversion of most of the valencene and other sesquiterpene volatiles into the terpenoid pathway, together with the high production of apocarotenoid volatiles, might have resulted in the lower concentration of carotenoids, but high concentration of valencene observed in Temple [9]. No valencene deficiency has been reported in other plant species. In our previous study, we assayed valencene abundance in juice in a segregating population from the cross of Fortune and Murcott mandarins and mapped a dominant locus responsible for valencene production on a mandarin genetic map [10]. Here, we further investigated the components of sesquiterpene, the transcriptional and genomic differences, and finally identify a deletion in the promoter region of valencene synthase, probably responsible for severe reduction of *Cstps1* gene expression associated with biosynthesis of valencene in Murcott. The study provides new insights into the role of *Cstps1* and the biosynthesis of terpenes in citrus.

## Results

### Relationship between valencene production and *Cstps1* gene expression

Valencene and other sesquiterpenes were measured in mature fruit of Fortune and Murcott mandarins, as well as nine of their F1 progenies randomly selected from the previous mapping population. As shown in Table 1, FoMu097 and FoMu114 produced large amounts of valencene and other sesquiterpenes. Fortune, FoMu003 and FoMu007 produced relatively lower levels of valencene and sesquiterpenes, whereas, Murcott, FoMu001, FoMu005 and FoMu012 did not produce detectable levels of valencene or other sesquiterpenes. FoMu004 also did not produce valencene, but had very low levels of sesquiterpenes, equaling to 3.65% of the total amount of sesquiterpenes in Fortune. The production of valencene was detected in FoMu083 but severely reduced, which accounted for only 5.87% of the total sesquiterpenes, far less than 50–80% of the total sesquiterpenes observed in other genotypes with detectable valencene.

**Table 1:** Relative abundance of sesquiterpenes in the segregating population between Fortune and Murcott mandarin flesh when the total sesquiterpenes in Fortune was set as 100. Mean value ± standard error, *n* = 6

	FoMu-001	FoMu-003	FoMu-004	FoMu-005	FoMu-007	FoMu-012	FoMu-083	FoMu-097	FoMu-114	For	Mur
Valencene	0.00 ± 0.00	19.73 ± 2.50	0.00 ± 0.00	0.00 ± 0.00	45.72 ± 3.69	0.00 ± 0.00	3.38 ± 0.79	173.96 ± 22.99	240.86 ± 22.18	79.72 ± 14.63	0.00 ± 0.00
α-Cubebene	0.00 ± 0.00	0.64 ± 0.17	0.25 ± 0.10	0.00 ± 0.00	0.87 ± 0.13	0.00 ± 0.00	2.16 ± 0.64	6.88 ± 1.10	1.20 ± 0.49	0.00 ± 0.00	0.00 ± 0.00
β-Elemene	0.00 ± 0.00	0.65 ± 0.17	0.70 ± 0.18	0.00 ± 0.00	1.33 ± 0.12	0.00 ± 0.00	3.23 ± 0.53	8.39 ± 0.91	4.68 ± 0.50	0.00 ± 0.00	0.00 ± 0.00
Caryophyllene	0.00 ± 0.00	0.66 ± 0.17	0.18 ± 0.07	0.00 ± 0.00	1.26 ± 0.08	0.00 ± 0.00	0.66 ± 0.17	7.14 ± 0.96	8.13 ± 1.26	0.39 ± 0.12	0.00 ± 0.00
Spirolepechinene	0.00 ± 0.00	0.00 ± 0.00	0.00 ± 0.00	0.00 ± 0.00	1.02 ± 0.11	0.00 ± 0.00	0.00 ± 0.00	5.34 ± 0.89	4.15 ± 0.39	2.51 ± 0.50	0.00 ± 0.00
α-Humulene	0.00 ± 0.00	0.75 ± 0.20	0.18 ± 0.07	0.00 ± 0.00	0.13 ± 0.05	0.00 ± 0.00	1.69 ± 0.45	4.08 ± 0.31	2.77 ± 0.53	0.00 ± 0.00	0.00 ± 0.00
γ-Muurolene	0.00 ± 0.00	0.00 ± 0.00	0.00 ± 0.00	0.00 ± 0.00	0.00 ± 0.00	0.00 ± 0.00	0.00 ± 0.00	0.91 ± 0.24	1.61 ± 0.35	0.59 ± 0.18	0.00 ± 0.00
α-Farnesene	0.00 ± 0.00	0.50 ± 0.13	0.00 ± 0.00	0.00 ± 0.00	0.24 ± 0.06	0.00 ± 0.00	6.66 ± 1.27	12.58 ± 1.73	9.73 ± 2.16	0.00 ± 0.00	0.00 ± 0.00
Germacrene D	0.00 ± 0.00	0.00 ± 0.00	0.00 ± 0.00	0.00 ± 0.00	0.00 ± 0.00	0.00 ± 0.00	0.00 ± 0.00	0.00 ± 0.00	0.00 ± 0.00	0.67 ± 0.19	0.00 ± 0.00
α-Muurolene	0.00 ± 0.00	0.71 ± 0.19	0.11 ± 0.05	0.00 ± 0.00	0.00 ± 0.00	0.00 ± 0.00	2.36 ± 0.59	2.28 ± 0.35	0.00 ± 0.00	3.29 ± 0.82	0.00 ± 0.00
α-Selinene	0.00 ± 0.00	1.26 ± 0.25	0.00 ± 0.00	0.00 ± 0.00	2.99 ± 0.26	0.00 ± 0.00	2.99 ± 0.58	13.01 ± 1.57	15.95 ± 1.76	7.03 ± 1.66	0.00 ± 0.00
Premnaspirodiene	0.00 ± 0.00	0.00 ± 0.00	0.00 ± 0.00	0.00 ± 0.00	0.87 ± 0.16	0.00 ± 0.00	0.00 ± 0.00	0.00 ± 0.00	5.42 ± 0.46	2.82 ± 0.78	0.00 ± 0.00
δ-Cadinene	0.00 ± 0.00	11.33 ± 1.70	1.92 ± 0.43	0.00 ± 0.00	3.51 ± 0.57	0.00 ± 0.00	28.25 ± 3.87	39.49 ± 3.56	19.17 ± 3.18	0.22 ± 0.06	0.00 ± 0.00
Calamenene	0.00 ± 0.00	2.03 ± 0.40	0.28 ± 0.11	0.00 ± 0.00	0.79 ± 0.15	0.00 ± 0.00	3.57 ± 0.81	5.35 ± 1.01	5.80 ± 0.79	0.06 ± 0.03	0.00 ± 0.00
7-epi-α-Selinene	0.00 ± 0.00	0.00 ± 0.00	0.00 ± 0.00	0.00 ± 0.00	2.22 ± 0.22	0.00 ± 0.00	0.00 ± 0.00	3.08 ± 0.49	9.27 ± 0.79	2.45 ± 0.59	0.00 ± 0.00
(E)-Cadina-1,4-diene	0.00 ± 0.00	0.00 ± 0.00	0.00 ± 0.00	0.00 ± 0.00	0.11 ± 0.04	0.00 ± 0.00	0.29 ± 0.12	2.11 ± 0.35	0.94 ± 0.28	0.00 ± 0.00	0.00 ± 0.00
(E)-Nerolidol	0.00 ± 0.00	0.00 ± 0.00	0.00 ± 0.00	0.00 ± 0.00	0.00 ± 0.00	0.00 ± 0.00	2.08 ± 0.60	0.00 ± 0.00	0.00 ± 0.00	0.00 ± 0.00	0.00 ± 0.00
Cubenol	0.00 ± 0.00	1.32 ± 0.27	0.00 ± 0.00	0.00 ± 0.00	0.08 ± 0.03	0.00 ± 0.00	0.18 ± 0.07	0.74 ± 0.14	0.20 ± 0.08	0.00 ± 0.00	0.00 ± 0.00
Total	0	39.57	3.64	0	61.12	0	57.51	285.33	329.88	100	0
Valencene % of total	NA	49.86	0	NA	74.81	NA	5.87	60.97	73.01	79.72	NA

**Fig. 2:**
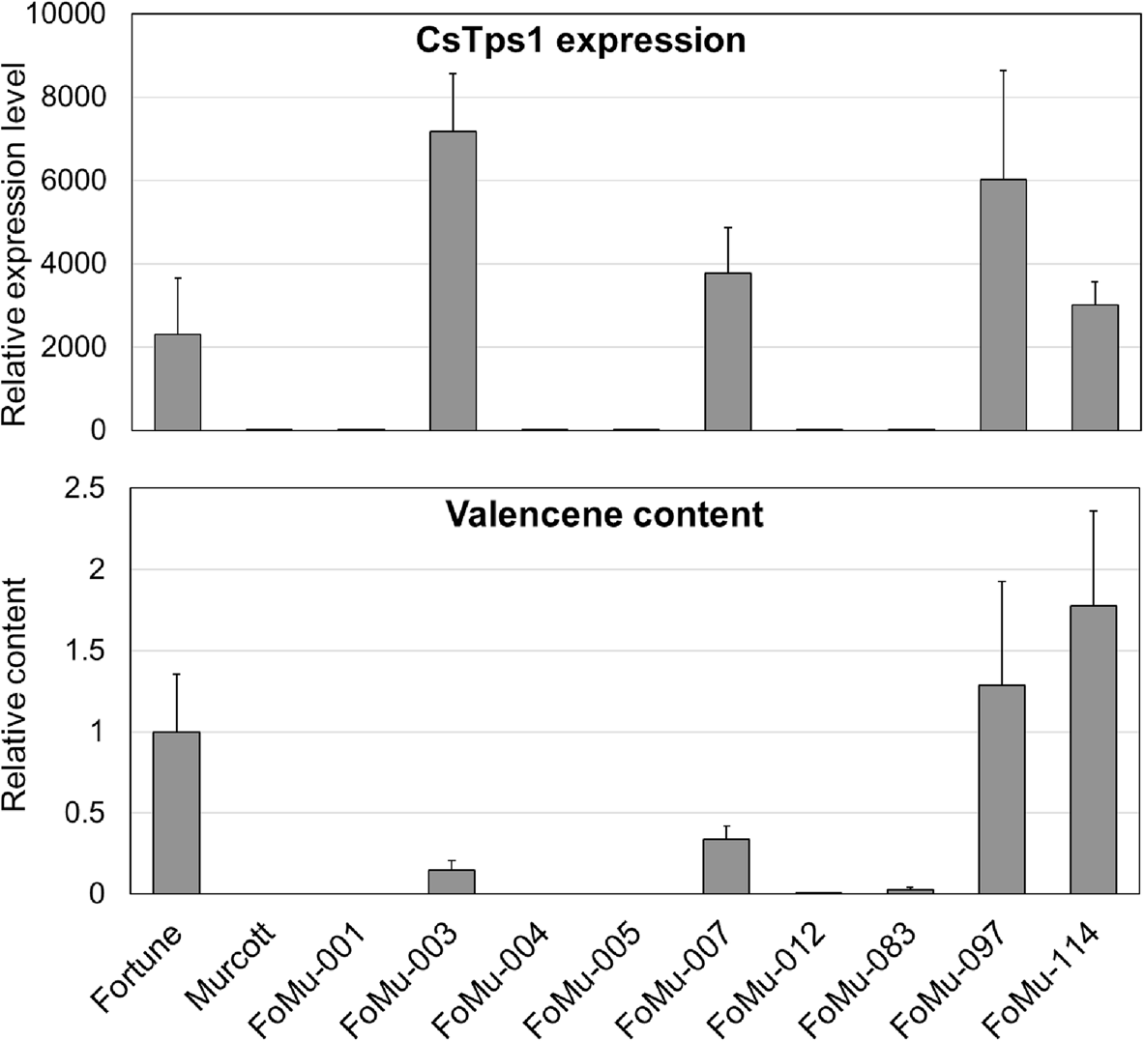
Valencene content and *Cstps1* expression levels among Fortune (For), Murcott (Mur) and their progenies (FoMu-001, FoMu-003, FoMu-004, FoMu-005, FoMu-007, FoMu-012, FoMu-083, FoMu-097 FoMu-114)

In our previous study, we found that the gene expression of *Cstps1* in mature fruit was over 2720 times higher in mandarin hybrid Temple than in Murcott [9]. Murcott mandarin *Cstps1* gene expression is severely reduced. Phenotypic segregation was determined from 92 individuals and was 45:47 (non-detected: valencene), not significantly different from a 1:1 ratio (χ2 = 0.134) (Additional file 1: Table S1). To determine if *Cstps1* is the gene underlying the quantitative trait loci (QTL) controlling valencene content, we analyzed the expression level of *Cstps1* in peel of ripe fruits to determine valencene content from nine selected progenies, as well as the two parents (Fig. 2). This analysis showed high levels of *Cstps1* expression in Fortune and the four selected progenies with abundant valencene, but no detectable expression in Murcott and the four selected progenies without valencene, as well as FoMu083 with very low level of valencene. The presence or absence of *Cstps1* gene expression is mostly consistent with the presence or and absence of valencene, although there is no clear relationship between *Cstps1* gene expression levels and amounts of valencene produced.

### Isolation and comparison of *Cstps1* and the promoter regions in fortune and Murcott

**Fig. 3:**
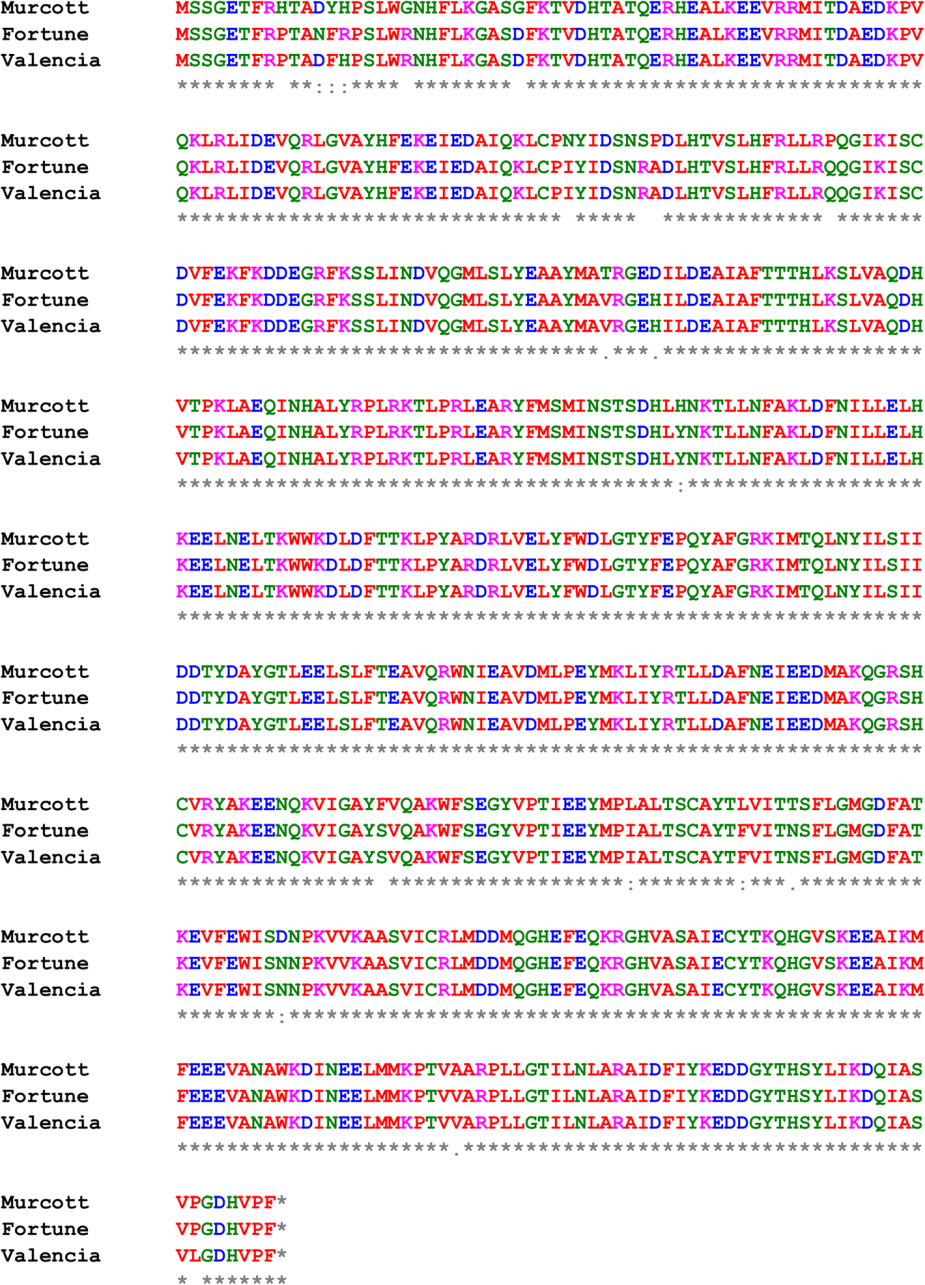
Multiple alignment of deduced amino acid sequences of *Cstps1* among two mandarin hybrid varieties, Fortune and Murcott, and Valencia, a sweet orange (gi:572152984)

QTL analysis identified one dominant locus associated with valencene abundance, which had a LOD value of 17.2 and explained the phenotypic variance as high as 59.8% [10]. The identified QTL was linked to two adjacent marker m66_s3 (21.0 cM) and m261_s3 (25.9 cM) on Fortune genetic linkage group 3 (Additional file 2: Table S2), and the two markers were both located on Scaffold_3 of Clementine mandarin genome. The valencene synthase gene *Cstps1*, previously isolated and characterized in Valencia sweet orange, was also located on scaffold_3 of Clementine mandarin genome. Based on *Cstps1* sequence information, cDNA and the genomic DNA region of *Cstps1* gene were isolated from Fortune and Murcott fruit. They both contain a 1647 bp open reading frame, encoding 548 amino acids. Multiple alignment analysis of the deduced protein sequence from *Cstps1* gene (Fig. 3) indicated that Fortune showed an almost identical protein sequence to the known valencene synthase protein in Valencia orange (99.5%), whereas Murcott exhibited a 96.7% identity. No clear differences were found in estimated Cstps1 protein structure between Fortune and Murcott using a protein program (http://www.sbg.bio.ic.ac.uk/phyre2/). By analyzing the active sites and binding sites on the protein of Cstps1 among Murcott, Fortune and Valencia, we found that except for only one active site (8–15 aa), all others are highly conserved among three varieties (Additional file 3: Figure S1).

**Fig. 4:**
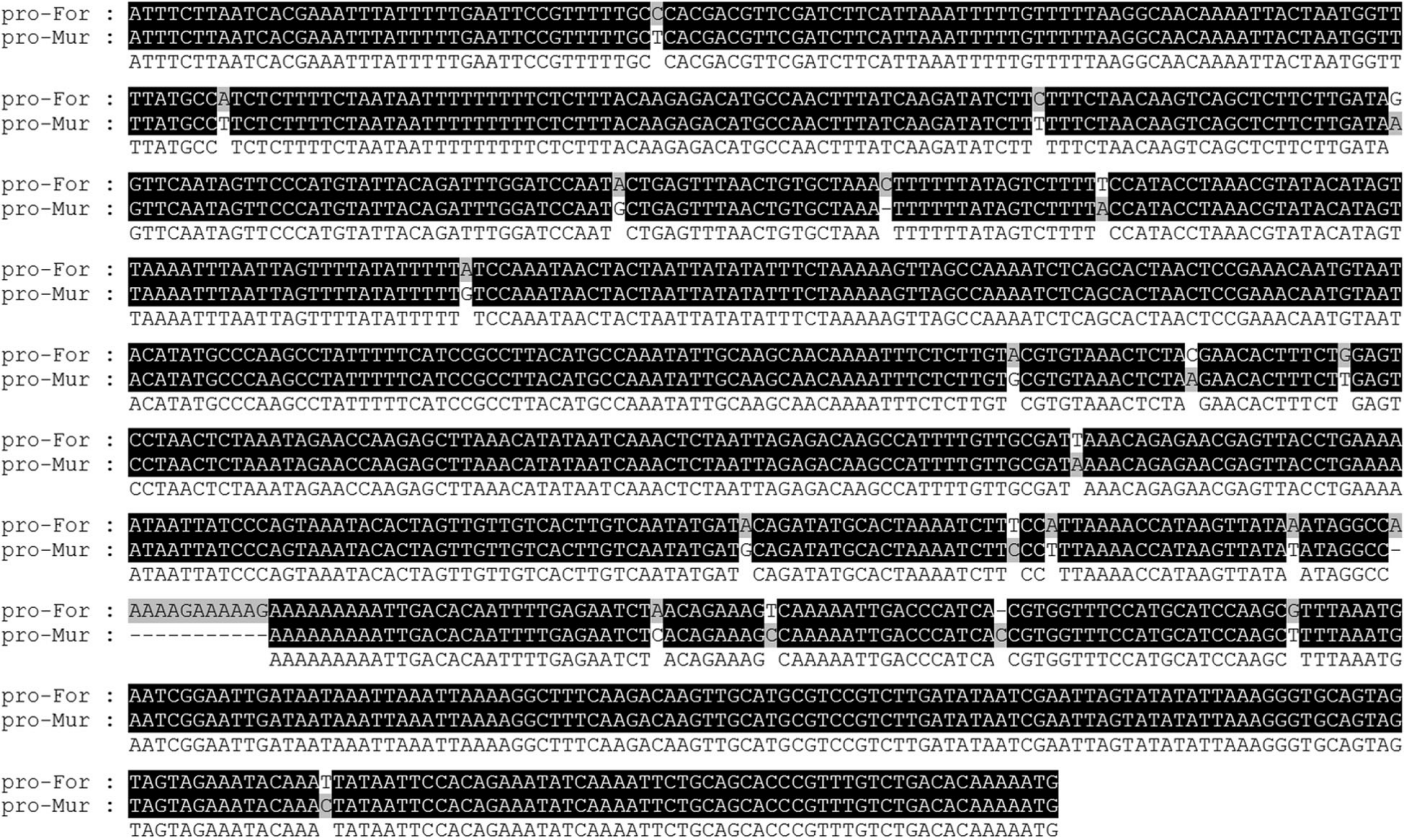
Alignment of *Cstps1* promoter sequences for Fortune and Murcott. The upstream regulatory regions of *Cstps1* were isolated by genome walking in Fortune and Murcott. Sequence alignment showed a deletion of 12 nucleotides (GAAAAAGAAAAA) nearby the *Cstps1* core promoter region in the Murcott compared to Fortune

**Fig. 5:**
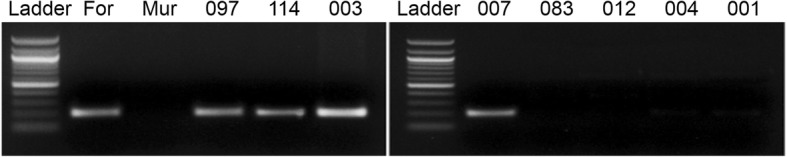
PCR-based marker for a 12-nucleotide deletion allele in *Cstps1* promoter among Fortune (For), Murcott (Mur) and their progenies (FoMu-097, FoMu-114, FoMu-003, FoMu-007, FoMu-083, FoMu-004, FoMu-001, FoMu-012); ladder is 100 bp DNA. The result exactly matches the gene expression of *Cstps1* in these individuals

Due to severely reduced *Cstps1* gene expression associated with undetectable valencene production, we investigated whether changes in the *Cstps1* promoter region could cause the presence or absence of *Cstps1* expression. We further cloned the promoter region of *Cstps1* gene based on genome walking. A total length of 969 bp nucleotides upstream of the *Cstps1* gene was cloned from Fortune, while 957 bp nucleotides were cloned from Murcott. Alignment analysis of the promoter sequence between Fortune and Murcott indicated extremely high identity to each other, except for a deletion of 12 nucleotides in Murcott located − 270 bp upstream of *Cstps1* gene. The nucleotide sequence of the 12-bp deletion in Murcott could be either of five types due to sequence alignment: 5′-AAAAAGAAAAAG-3′, 5′-AAAAGAAAAAGA-3′, 5′-AAAGAAAAAGAA-3′, 5′-AAGAAAAAGAAA-3′, 5′-AGAAAAAGAAAA-3′, or 5′-GAAAAAGAAAAA-3′ (Fig. 4). The presence or absence of the 12 nucleotides in Fortune, Murcott and randomly selected progenies was further confirmed by PCR with specific primers (Fig. 5). As shown, the presence or absence of these 12 nucleotides in the *Cstps1* promoter among these materials was consistent with the presence or absence of the *Cstps1* gene expression.

### Functional analysis of *Cstps1* promoter in sweet orange leaves

**Fig. 6:**
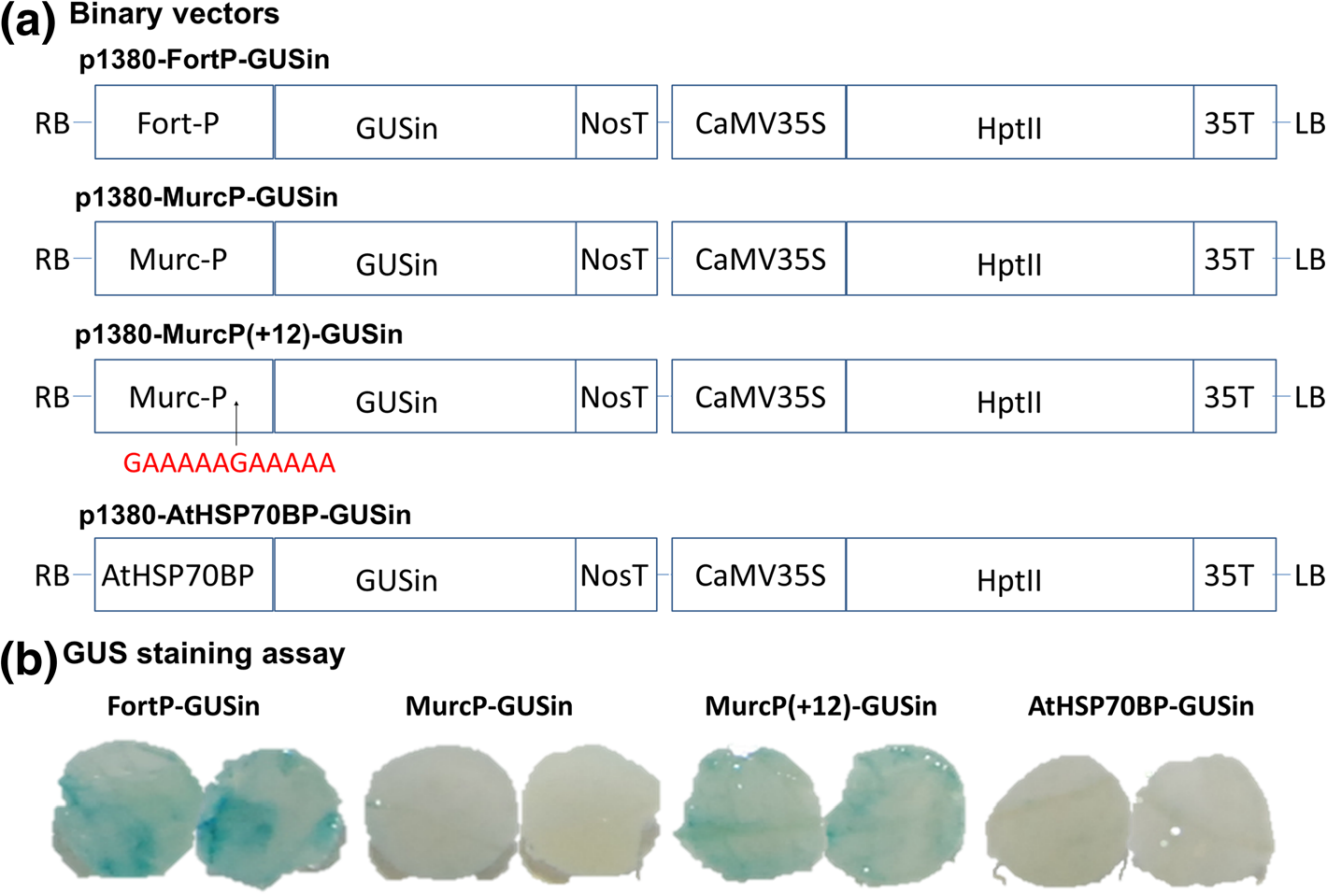
Transient expression analysis of *Cstps1* promoter in sweet orange leaves. (**a**) Binary vectors used for agroinfiltration. Schematic diagram of binary plasmid used in this study: AtHSP70BP is the Arabidopsis heat shock-regulated protein 70B promoter. Fort-P is Fortune promoter. Murc-P is Murcott promoter. Murc-P (+ 12) is recovered by re-inserting the 12 bp deletion into the Murcott promoter. The 12 nucleotides are highlighted by red. GUSin, the intron-containing β-glucuronidase; NosT, the nopaline synthase gene terminator; HptII, the coding sequence of hygromycin phosphotransferase II; LB and RB, the left and right borders of the T-DNA region; (**b**) Via *Xanthomonas citri* subsp. citri (Xcc)-facilitated agroinfiltration in Valencia orange leaves with blue color showing GUS expression for Fortune *Cstps1* promoter (FortP-GUSin, 975 bp), Murcott *Cstps1* promoter (MurcP-GUSin, 957 bp), Murcott *Cstps1* promoter + the missing 12-bp (MurcP(+ 12)-GUSin, and AtHSP70BP-GUSin (Arabidopsis heat shock-regulated protein 70B promoter). The experiment was repeated twice with similar results (data not shown)

We hypothesized that the 12-nucleotide deletion in the promoter region of the Murcott *Cstps1* gene might be the reason for severely reduced *Cstps1* gene expression and the resulting lost production of valencene synthase in Murcott. To test the hypothesis, *Xanthomonas citri* subsp. citri (Xcc)-facilitated agroinfiltration was used to study *Cstps1* promoter function by a GUS transient expression assay using binary vectors p1380-FortP-GUSin, p1380-MurcP-GUSin and and p1380-MurcP(+ 12)-GUSin (Fig. 6a). The negative control was p1380-AtHSP70BP-GUSin. After Xcc-facilitated agroinfiltration, GUS was detected by histochemical staining (Fig. 6b). Our previous study indicated that Xcc-facilitated agroinfiltration enhances gene expression in citrus leaves using binary vector p1380-35S-GFP and p1380-35S-GUSin [11]. Notably, the Fortune *Cstps1* promoter (969 bp) showed significant GUS expression, but the Murcott *Cstps1* promoter (957 bp) did not. However, the function of the Murcott derived promoter could be mostly recovered by re-inserting the 12-bp deletion (p1380-MurcP(+ 12)-GUSin). (Fig. 6b). These results indicate that the deletion of the 12 nucleotides, to a large extent, caused absence of *Cstps1* gene expression.

## Discussion

Valencene is the major sesquiterpene in citrus oil. Sweet orange has much more abundant valencene than other citrus species. Its concentration increases as fruits become more mature and the flavor quality also increases [5, 9]. Because of this, valencene can be used as an index of citrus maturity [9]. In our previous study, we found the expression of *Cstps1* to be severely reduced in Murcott mandarin, and the product of *Cstps1*, valencene, to be undetectable [9]. This reduction of valencene has been reported previously and was associated with higher carotenoid concentration in mandarin hybrid fruits [9], and positively correlated with other volatiles. In this study, individual FoMu083 with absence of *Cstps1* gene expression still had detectable valencene, but its 5.87% of total sesquiterpenes was much lower than 70% of total sesquiterpenes normally found in citrus varieties from a previous study [12]. Bai et al. [12] indicated that there was a high correlation between valencene and total sesquiterpene production.

Engineering terpene metabolism in plants was not simple [13]. Heterologous expression of *Cstps1* from the sweet orange alone was not sufficient to enable valencene production, possibly due to the low amount of farnesyl pyrophosphate (FPP) by endogenous prenyltransferases [14, 15]. A previous study [5] showed that valencene could accumulate up to a maximum of ~ 60 μg per fresh weight of flavedo tissue, which was much lower than the maximum expectation of valencene production. The expression of *Cstps1* gene can be increased five- to ten-fold by the exogenous application of the growth regulator ethylene, however, valencene accumulation was not increased to the same extent. Sharon-Asa et al. [5] suspected that valencene is synthesized and accumulates in specific juice glands or sacs, which probably limit their maximal functioning production capacity. Therefore, attempts at overexpressing *Cstps1* in plants to increase valencene content could not be easily achieved.

Sesquiterpene synthase encompassed the largest number of putative full length *Cstps*, despite their low contribution to total volatiles (less than 18%) in sweet orange [15]. Alquezar et al. [16] identified 25 sesquiterpenes, and Cstps1 was a protein virtually identical (99.64%) to Cs5g12900.2, there being only two amino acid changes located at the N-terminus. In this study, we found 18 sesquiterpenes (Table 1). There was no clear relationship between valencene production and the *Cstps1* gene expression level among *Cstps1*-expressing samples (Table 1 and Fig. 2). We suspect that other genes in the terpene biosynthesis pathway might play roles as well. Valencene is generally recognized as the major product produced by *Cstps1* from farnesyl pyrophosphate, an intermediate of carotenoid biosynthesis [5], although Germacrene A was found as a major side product from the *C. sinensis* valencene synthase. [17]. Monoterpenes, sesquiterpenes and diterpenes appear as relatively simple branch pathways of the MVA and MEP pathways (Fig. 1). Furthermore, Yu et al. [10] found that valencene was correlated with other monoterpenes.

The segregating F1 population of Fortune × Murcott showed that the segregation of valencene production in fruit exhibited an inheritance Mendelian ratio of 1:1 (χ2 = 0.134) (Additional file 1: Table S1). The valencene accumulation was inherited as a simple Mendelian locus, which was mapped on Fortune linkage group 3 (Additional file 2: Table S2). We suspect there might be natural recessive alleles in the *Cstps1-*associated phenotype. In strawberry, mesifurane and γ-decalactone were mapped as single Mendelian traits [16]. Based on Clementine mandarin genome data from Phytozome v1.0 (https://phytozome.jgi.doe.gov), the *Cstps1* gene was located on Scaffold_3 and not far away from the linked markers confirming the previous finding [5] that *Cstps1* controls the production of valencene in citrus.

Shen et al. (2016) [18] found that the expression pattern of *CitAP2.10* was positively correlated with valencene content and *Cstps1* expression and suggested that CitAP2.10 could trans-activate the *Cstps1* promoter. This suggests that regulatory elements are required for regulation of *Cstps1*. However, *CitAP2.10* are located in chromosome 4 and 6 based on genome sequencing data from Phytozome v1.0 http://www.phytozome.net (Phytozome: Cclementina 182 v1.fa.gz). We did not find QTLs in linkage group 4 and 6 associated with valencene production in our segregating population. The discrepancy between identified QTLs in a genetic map and physical location of *Cstps1* and *CitAP2.10* in the Clementine genome suggest that the relationship between a physical map and genetic map are not always direct. The amount of recombination between any two equidistant markers can vary significantly through the genome in genetic map [19]. In addition, valencene production is a complex phenomenon, and might involve substrate availability or other genes in the metabolic pathway.

Cloning and comparison of the upstream regulatory promoter regions of *Cstps1* gene revealed certain differences in previously identified cis-acting elements between Murcott and Fortune. In this study, we characterized a deletion of 12 nucleotides in a core promoter region associated with the loss of function and low expression of *Cstps1*, which resulted in an absence or dramatic reduction of valencene content. Citrus is a perennial tree and is difficult to transform. In this study, we used our previously developed method of *Xanthomonas citri* subsp. citri (Xcc)-facilitated agroinfiltration to promote transient protein expression in Valencia sweet orange leaves, which are recalcitrant to common agroinfiltration [11]. The current system using Xcc-facilitated agroinfiltration enhanced transient protein expression in citrus [11]. The possible mechanism to explain the fact that Xcc pre-treatment could significantly increase the transient expression of inoculated citrus leaves, is that the PthA4 effector is known to be translocated from Xcc into plant cell nuclei, where it activates downstream target genes [11]. Thus, transient expression experiments were conducted to validate the role of the 12-nucleotide deletion in a promoter region of *Cstps1*. Although the mutation presented in this paper is most likely responsible for the deficient allele, the possibility of further sequence mutations in the coding region contributing to the lost function of *Cstps1* and the consequent absence of valencene could not be ruled out at this point.

## Conclusions

Murcott mandarin hybrid might be a mutant with severely reduced gene expression of *Cstps1*. The deletion of the 12-nucleotides in the upstream regulatory promoter regions of the *Cstps1* gene could be a crucial switch of *Cstps1* transcription. Future studies are needed to confirm the functional differences in *Cstps1* and promoter regions in other citrus species in order to further understanding of valencene biosynthesis in citrus.

## Methods

### Plant materials

Fortune and Murcott mandarins (*Citrus reticulata* Blanco), as well as nine Fortune × Murcott hybrids were used in this study. Fortune produces abundant valencene, whereas valencene was undetectable in Murcott. The nine hybrids were randomly selected from a F1 segregated population for genetic mapping [11], including FoMu-001, FoMu-003, FoMu-004, FoMu-005, FoMu-007, FoMu-012, FoMu-083, FoMu-097, FoMu-114. Fruits with uniform size and color, and free of peel defects from Fortune, Murcott and F1 hybrids were harvested randomly in their estimated commercial maturity in 2012 and 2013. The production of valencene is maximized when the fruit are mature. All samples were stored in − 80 °C for future experiments.

### Volatile compound identification

Sample preparation for volatile and aroma identification used the same methods as previously described [10]. Briefly, 3 mL juice was mixed with the same volume of saturated sodium chloride solution (359 g/L), and an internal standard 3-hexanone with a final concentration of 10 μM. The mixture was prepared in a 20 ml glass vial and sealed with a silicone/PTFE septum. The vials were stored at − 20 °C until analyzed. For analysis, juice samples were incubated for 30 min at 40 °C, and a 2.0 cm solid phase microextraction (SPME) fiber (50/30 μm DVB/Carboxen/PDMS; Supelco, Bellefonte, PA) was used to extract the volatiles. Volatiles were analyzed by a GCMS (Model 6890/5973 N, Agilent, Santa Clara, CA) with a DB5 column (60-m length, 0.25-mm i.d., 1.00-μm film thickness). The program settings were according to the report [10]. Volatile compounds were identified by comparing their mass spectra with the authorized standard chemicals, the NIST mass spectral database, and published retention indices. The amount of each aroma volatile was expressed as relative content (aroma volatile peak area over internal standard peak area).

### RNA extraction and QRT-PCR

Total RNA was extracted from the fruit peel of each sample using the PureLink plant RNA reagent (Thermo Fisher Scientific) according to the manufacturer’s instructions. DNA was removed by the Turbo DNA-free Kit (Ambion, Austin, TX). The Brilliant III Ultra-Fast SYBR Green QRT-PCR Master Mix (Agilent Technology) was used for QRT-PCR. It was carried out in the Agilent Mx3005P System (Agilent Technology) using glyceraldehyde 3-phosphate dehydrogenase gene (GAPDH) as a reference gene. The specific primers for QRT-PCR of *Cstps1* were designed according to the report of Sharon-Asa et al. (2003) [5]. The results of relative *Cstps1* expression were expressed as normalized mean values and standard error.

### Isolation of *Cstps1* cDNA and promoters

Total RNA of Fortune and Murcott mandarin fruit were used to synthesize cDNA using the Revert Aid First Strand cDNA Synthesis Kit (Thermo Fisher Scientific). The primers (Tps1-F: 5′-ATGTCGTCTGGAGAAACATTTC-3′; Tps1-R: 5′-TCAAAATGGAACGTGGTCTCCT-3′) for the amplification of *Cstps1* cDNA were designed based on the sequence of *Citrus sinensis TPS1* from NCBI (gi:572152984). PCR products were cloned into pGEM-T vector (Promega Corporation) for sequencing. Genomic DNA was extracted with DNeasy Plant Kits (Qiagen, Valencia, CA) from the leaves of Fortune and Murcott mandarin. Genomic region of *Cstps1* gene was amplified by PCR using the same primers for the isolation of cDNA, and PCR products were cloned into pGEM-T vector for sequencing. The 5′ upstream region of *Cstps1* was isolated using GenomeWalker Universal Kit (Clontech Laboratories, Mountain View, CA). Genomic DNA was digested by the four restriction enzymes (DraI, EcoRV, PvuII and StuI) to build blunt end GenomeWalker libraries. Using the libraries as template, primary and nested PCRs were performed using the GenomeWalker adaptor primers (AP1: 5′-GTAATACGACTCACTATAGGGC-3′; AP2: 5′-ACTATAGGGCACGCGTGGT-3′) provided in the kit and gene-specific primers (GSP1: 5′-GGGATGTTTGGGTTCATCTTTAC-3′; GSP2: 5′-CAGAAGCACCTTTGAGGAAATG-3′) designed according to the obtained sequences of *Cstps1* gene. Nested PCR products were checked by electrophoresis in 1.5% (*w*/*v*) agarose gel, and the products with reasonable size were cloned into the pGEM-T vector for sequencing. For all times of sequencing, at least five clones were randomly selected and sequenced by the Interdisciplinary Center for Biotechnology sequencing facility of the University of Florida. The software SeqMan in DNAStar was used to align and analyze the sequences. The inconsistent single nucleotide sites with ratio of base type above or equal to 1/3 were replaced with corresponding degenerate nucleotide codes, otherwise, they were considered as sequencing errors. The program ClustalW2 in European Bioinformatics Institute were used to process the output sequences and construct alignment between different varieties.

### Plasmid construction

Using a pair of primers, FMP1-SbfI (5′- AGGTCCTGCAGGATCACGAAATTTATTTTTGAATTCCG-3′) and FMP2-BglII (5′- AGGTAGATCTTTTGTGTCAGACAAACGGGTGCTGC-3′), the *Cstps1* promoter (P) was amplified from genomic DNA of Fortune and Murcott. After sequencing, the PCR products were digested with *Sbf*I and *Bgl*II, and inserted into *Sbf*I-*Bam*HI-treated p1380-35S-GUSin to form binary vectors p1380-FortP-GUSin, p1380-MurcP-GUSin (Fig. 6b). One part of the *Cstps1* promoter of Murcott was amplified using primers FMP1-SbfI and Murc(+ 12) P2 (5′-phosphorylated-CTTTTTGGCCTATTTATAACTTATGGTTTT-3′). After sequencing, the PCR products were digested with *Sbf*I. Another part was amplified using primers Murc(+ 12) P1 (5′-phosphorylated- AAAAAGAAAAAAAAATTGACACAATTTTGAGAA -3′) and FMP2-BglII. After sequencing, the PCR products were digested with *Bgl*II. Through three-way ligation, *Sbf*I-digested part of the *Cstps1* promoter and *Bgl*II-treated other part were inserted into *Sbf*I-*Bam*HI-cut p1380-35S-GUSin to form p1380-MurcP(+ 12)-GUSin (Fig. 6a). Binary vector p1380-AtHSP70BP-GUSin was used as negative control, which was developed previously [11]. It should be noted that all binary vectors harbor right border (RB) and left border (RB), which delimit transfer DNA (T-DNA). Agrobacterium strains transfer T-DNA to plant cells. In addition, the intron-containing GUS (GUSin) was employed here, since the intron is only spliced in plant cells, resulting in GUS enzymatic activity [20]. By the freeze-thaw method, the binary vectors were introduced into *A. tumefaciens* strain EHA105, respectively. Recombinant *Agrobacterium* cells were cultivated for Xcc-facilitated agroinfiltration.

### Xcc-facilitated agroinfiltration in Valencia sweet orange

Valencia plants, grown in a greenhouse at temperatures ranging from 25 to 30 °C, were pruned to produce uniform shoots before Xcc-facilitated agroinfiltration. Xcc-facilitated agroinfiltration in citrus leaves was performed as described previously with some modifications [11]. Briefly, citrus leaves were subjected for inoculation with a culture of actively growing XccΔgumC, which was re-suspended in sterile tap water (5 × 10^8^ CFU/ml). Thirty-six hours later, *Agrobacterium* cells containing p1380-FortP-GUSin, p1380-MurcP-GUSin, p1380-MurcP(+ 12)-GUSin or p1380-AtHSP70BP-GUSin, were agroinfiltrated into the same leaf area (Fig. 6b). Four days after agroinfiltration, leaves were assayed by the histochemical staining of GUS.

## Additional files

Additional file 1: **Table S1**. Valencene content in Fortune, Murcott, and their F1 progeny. (DOCX 16 kb)

Additional file 2: **Table S2**. QTLs associated with valencene content detected in the F1 population of Fortune × Murcott. (DOCX 13 kb)

Additional file 3: **Figure S1**. Prediction of active sites and binding sites on the protein of *Cstps1* among Murcott, Fortune and Valencia. (DOCX 25 kb)

## Abbreviations

Cstps1: Valencene synthase
DMAPP: Dimethylallyl pyrophosphate
FPP: Farnesyl pyrophosphate
FPPS: FPP synthase
G3P: Glyceraldehyde-3-phosphate
GGPP: Geranylgeranyl pyrophosphate
GGPPS: GGPP synthase
GPP: Geranyl pyrophosphate
GPPS: GPP synthase
IPP: Isopentenyl pyrophosphate
MEP: Methylerythritol phosphate
MVA: Mevalonic acid
QRT-PCR: Quantitative real-time Reverse transcription polymerase chain reaction
TPS: Terpene synthase
Xcc: *Xanthomonas citri* subsp. citri
