# Supplementary material for: Deficiency of valencene in mandarin hybrids is associated with a deletion in the promoter region of the valencene synthase gene
Source: BMC Plant Biol. 2019 Mar 13;19:101. doi: 10.1186/s12870-019-1701-6 (PMC6417135; doi:10.1186/s12870-019-1701-6)
Supplement: Supplementary file 1 — Table S1. Valencene content in Fortune, Murcott, and their F1 progeny. (DOCX 16 kb) [file 12870_2019_1701_MOESM1_ESM.docx]

| **Supplementary Table S1.** Valencene content in Fortune, Murcott, and their F1 progeny | | | |
| --- | --- | --- | --- |
| **Genotype** | **Rows** | **Mean Valencene** | **Mean Sesquiterpene** |
| FoMu-001 | 4 | 0 | 0 |
| FoMu-002 | 4 | 0 | 0.000606421 |
| FoMu-003 | 6 | 0.027028062 | 0.054209013 |
| FoMu-004 | 6 | 0 | 0.004984729 |
| FoMu-005 | 6 | 0 | 0 |
| FoMu-006 | 5 | 0 | 0.010278757 |
| FoMu-007 | 6 | 0.062631488 | 0.083724546 |
| FoMu-008 | 6 | 0 | 0 |
| FoMu-009 | 6 | 0 | 0 |
| FoMu-012 | 4 | 0 | 0 |
| FoMu-016 | 3 | 0 | 0.000312636 |
| FoMu-017 | 6 | 0.032378843 | 0.038644034 |
| FoMu-020 | 5 | 0.026116184 | 1.237906135 |
| FoMu-021 | 1 | 0.261940049 | 0.293760413 |
| FoMu-022 | 1 | 0.076878326 | 0.097406083 |
| FoMu-023 | 6 | 0 | 0.002111902 |
| FoMu-025 | 5 | 0 | 0.007784747 |
| FoMu-026 | 6 | 0.046684038 | 0.06669672 |
| FoMu-027 | 6 | 0.080854816 | 0.138153774 |
| FoMu-029 | 6 | 0.019130686 | 0.01965481 |
| FoMu-030 | 4 | 0.405177443 | 0.490426655 |
| FoMu-031 | 7 | 0.022957137 | 0.75068905 |
| FoMu-032 | 6 | 0 | 0.014128338 |
| FoMu-034 | 6 | 0.037668676 | 0.047723509 |
| FoMu-035 | 6 | 0.06124137 | 0.29554968 |
| FoMu-036 | 4 | 0 | 0.005371526 |
| FoMu-037 | 6 | 0.970844654 | 1.443855794 |
| FoMu-039 | 5 | 0 | 0 |
| FoMu-040 | 3 | 0.005914199 | 0.067438461 |
| FoMu-041 | 1 | 0.007325397 | 0.016987267 |
| FoMu-045 | 6 | 0.019845981 | 0.737058713 |
| FoMu-046 | 5 | 0 | 0.000579191 |
| FoMu-048 | 6 | 0.222838696 | 0.261967003 |
| FoMu-051 | 6 | 0 | 0.001733668 |
| FoMu-053 | 9 | 0.051380112 | 0.057464646 |
| FoMu-054 | 1 | 0 | 0 |
| FoMu-057 | 2 | 0 | 0.000856806 |
| FoMu-059 | 3 | 0.007817271 | 0.010855559 |
| FoMu-060 | 4 | 0 | 0.002025181 |
| FoMu-061 | 6 | 0.125989777 | 0.179848126 |
| FoMu-062 | 3 | 0 | 0.001425414 |
| FoMu-063 | 4 | 0 | 0 |
| FoMu-065 | 6 | 0.020954228 | 0.024001548 |
| FoMu-066 | 4 | 0.033642273 | 0.039142587 |
| FoMu-067 | 6 | 0 | 0.000249269 |
| FoMu-068 | 6 | 0.304617076 | 0.541922633 |
| FoMu-069 | 5 | 0.780290613 | 1.001308185 |
| FoMu-070 | 6 | 0 | 0 |
| FoMu-071 | 6 | 0.320835356 | 0.490378155 |
| FoMu-072 | 4 | 0 | 0 |
| FoMu-074 | 6 | 0.015043264 | 0.032498278 |
| FoMu-075 | 5 | 0.032706855 | 0.145519019 |
| FoMu-076 | 3 | 0.151630903 | 1.189929484 |
| FoMu-077 | 2 | 0.230954047 | 1.203074936 |
| FoMu-078 | 6 | 0.735356027 | 0.849091658 |
| FoMu-080 | 2 | 0.004218364 | 0.012257296 |
| FoMu-082 | 1 | 0.003085548 | 0.023309185 |
| FoMu-083 | 6 | 0.004626083 | 0.078785549 |
| FoMu-085 | 6 | 0 | 0 |
| FoMu-086 | 6 | 0.00625509 | 0.006414735 |
| FoMu-088 | 5 | 0.033163036 | 0.302208334 |
| FoMu-089 | 5 | 0 | 0.014078222 |
| FoMu-090 | 6 | 0 | 0.109496799 |
| FoMu-091 | 1 | 0.103319981 | 5.492559391 |
| FoMu-092 | 6 | 0 | 0 |
| FoMu-093 | 6 | 0 | 0 |
| FoMu-094 | 6 | 0 | 0.014661253 |
| FoMu-095 | 6 | 0 | 0.027618533 |
| FoMu-096 | 6 | 0 | 0 |
| FoMu-097 | 6 | 0.23830299 | 0.390868245 |
| FoMu-098 | 6 | 0 | 0.020734767 |
| FoMu-099 | 6 | 0.007951067 | 0.009154027 |
| FoMu-101 | 6 | 0.002583089 | 0.020021031 |
| FoMu-102 | 5 | 0.030754645 | 0.034897077 |
| FoMu-103 | 1 | 0.065258309 | 0.166095689 |
| FoMu-105 | 6 | 0.024286776 | 0.028322541 |
| FoMu-106 | 6 | 0.011831295 | 0.012203095 |
| FoMu-107 | 1 | 0 | 0.009416693 |
| FoMu-108 | 4 | 0 | 0.001151262 |
| FoMu-109 | 6 | 0.08817828 | 0.106902729 |
| FoMu-110 | 6 | 0 | 0.00309912 |
| FoMu-111 | 3 | 0 | 0.00089236 |
| FoMu-112 | 5 | 0 | 0.000551553 |
| FoMu-114 | 6 | 0.329949044 | 0.451893559 |
| FoMu-115 | 3 | 0.416549663 | 2.383811361 |
| FoMu-116 | 4 | 0 | 0.001284277 |
| FoMu-119 | 6 | 0 | 0.000921988 |
| FoMu-120 | 3 | 0 | 0.005126172 |
| FoMu-122 | 6 | 0 | 0.006274193 |
| FoMu-123 | 1 | 0 | 0.024293364 |
| FoMu-126 | 2 | 0 | 0.006894999 |
| For | 12 | 0.109205546 | 0.136636323 |
| Mur | 6 | 0 | 0 |
|  |  |  |  |
