# Supplementary material for: Deficiency of valencene in mandarin hybrids is associated with a deletion in the promoter region of the valencene synthase gene
Source: BMC Plant Biol. 2019 Mar 13;19:101. doi: 10.1186/s12870-019-1701-6 (PMC6417135; doi:10.1186/s12870-019-1701-6)
Supplement: Supplementary file 2 — Table S2. QTLs associated with valencene content detected in the F1 population of Fortune × Murcott. (DOCX 13 kb) [file 12870_2019_1701_MOESM2_ESM.docx]

**Supplementary Table S2**. QTLs associated with valencene content detected in the F_1_ population of Fortune × Murcott using Composite Interval Mapping and Kruskal-Wallis (K-W) test. Only one QTL was found for all the sampling time points.

| QTL | Time | Location | K-W | Position | Max LOD | Nearest marker | Marker position | Effect |
| --- | --- | --- | --- | --- | --- | --- | --- | --- |
| PK284-3.3 | 2012 Jan | FOR3.3 | p< 10^-4^ | 23.0 cM | 3.51 | m66_s3 | 21.0 cM | 18.5% |
|  | 2012 Feb | FOR3.3 | p< 10^-7^ | 21.0 cM | 9.4 | m66_s3 | 21.0 cM | 48.1% |
|  | 2013 Jan | FOR3.3 | p< 10^-7^ | 25.9 cM | 17.22 | m261_s3 | 25.9 cM | 59.8% |
|  | 2013 Feb | FOR3.3 | p< 10^-7^ | 25.9 cM | 12.72 | m261_s3 | 25.9 cM | 53.7% |
