# Supplementary material for: Deficiency of valencene in mandarin hybrids is associated with a deletion in the promoter region of the valencene synthase gene
Source: BMC Plant Biol. 2019 Mar 13;19:101. doi: 10.1186/s12870-019-1701-6 (PMC6417135; doi:10.1186/s12870-019-1701-6)
Supplement: Supplementary file 3 — Figure S1. Prediction of active sites and binding sites on the protein of Cstps1 among Murcott, Fortune and Valencia. (DOCX 25 kb) [file 12870_2019_1701_MOESM3_ESM.docx]

Murcott MSSGETFRHTADYHPSLWGNHFLKGASGFKTVDHTATQERHEALKEEVRRMITDAEDKPV

Fortune MSSGETFRPTANFRPSLWRNHFLKGASDFKTVDHTATQERHEALKEEVRRMITDAEDKPV

Valencia MSSGETFRPTADFHPSLWRNHFLKGASDFKTVDHTATQERHEALKEEVRRMITDAEDKPV

******** **:::**** ******** ********************************

Murcott QKLRLIDEVQRLGVAYHFEKEIEDAIQKLCPNYIDSNSPDLHTVSLHFRLLRPQGIKISC

Fortune QKLRLIDEVQRLGVAYHFEKEIEDAIQKLCPIYIDSNRADLHTVSLHFRLLRQQGIKISC

Valencia QKLRLIDEVQRLGVAYHFEKEIEDAIQKLCPIYIDSNRADLHTVSLHFRLLRQQGIKISC

******************************* ***** ************* *******

Murcott DVFEKFKDDEGRFKSSLINDVQGMLSLYEAAYMATRGEDILDEAIAFTTTHLKSLVAQDH

Fortune DVFEKFKDDEGRFKSSLINDVQGMLSLYEAAYMAVRGEHILDEAIAFTTTHLKSLVAQDH

Valencia DVFEKFKDDEGRFKSSLINDVQGMLSLYEAAYMAVRGEHILDEAIAFTTTHLKSLVAQDH

**********************************.***.*********************

Murcott VTPKLAEQINHALYRPLRKTLPRLEARYFMSMINSTSDHLHNKTLLNFAKLDFNILLELH

Fortune VTPKLAEQINHALYRPLRKTLPRLEARYFMSMINSTSDHLYNKTLLNFAKLDFNILLELH

Valencia VTPKLAEQINHALYRPLRKTLPRLEARYFMSMINSTSDHLYNKTLLNFAKLDFNILLELH

****************************************:*******************

Murcott KEELNELTKWWKDLDFTTKLPYARDRLVELYFWDLGTYFEPQYAFGRKIMTQLNYILSII

Fortune KEELNELTKWWKDLDFTTKLPYARDRLVELYFWDLGTYFEPQYAFGRKIMTQLNYILSII

Valencia KEELNELTKWWKDLDFTTKLPYARDRLVELYFWDLGTYFEPQYAFGRKIMTQLNYILSII

************************************************************

Murcott DDTYDAYGTLEELSLFTEAVQRWNIEAVDMLPEYMKLIYRTLLDAFNEIEEDMAKQGRSH

Fortune DDTYDAYGTLEELSLFTEAVQRWNIEAVDMLPEYMKLIYRTLLDAFNEIEEDMAKQGRSH

Valencia DDTYDAYGTLEELSLFTEAVQRWNIEAVDMLPEYMKLIYRTLLDAFNEIEEDMAKQGRSH

************************************************************

Murcott CVRYAKEENQKVIGAYFVQAKWFSEGYVPTIEEYMPLALTSCAYTLVITTSFLGMGDFAT

Fortune CVRYAKEENQKVIGAYSVQAKWFSEGYVPTIEEYMPIALTSCAYTFVITNSFLGMGDFAT

Valencia CVRYAKEENQKVIGAYSVQAKWFSEGYVPTIEEYMPIALTSCAYTFVITNSFLGMGDFAT

**************** *******************:********:***.**********

Murcott KEVFEWISDNPKVVKAASVICRLMDDMQGHEFEQKRGHVASAIECYTKQHGVSKEEAIKM

Fortune KEVFEWISNNPKVVKAASVICRLMDDMQGHEFEQKRGHVASAIECYTKQHGVSKEEAIKM

Valencia KEVFEWISNNPKVVKAASVICRLMDDMQGHEFEQKRGHVASAIECYTKQHGVSKEEAIKM

********:***************************************************

Murcott FEEEVANAWKDINEELMMKPTVAARPLLGTILNLARAIDFIYKEDDGYTHSYLIKDQIAS

Fortune FEEEVANAWKDINEELMMKPTVVARPLLGTILNLARAIDFIYKEDDGYTHSYLIKDQIAS

Valencia FEEEVANAWKDINEELMMKPTVVARPLLGTILNLARAIDFIYKEDDGYTHSYLIKDQIAS

**********************.*************************************

Murcott VPGDHVPF*

Fortune VPGDHVPF*

Valencia VLGDHVPF*

* *******

**Supplementary Figure S1** Prediction of active sites and binding sites on the protein of Cstps1 among Murcott, Fortune and Valencia. Gray indicates active site, while yellow indicates substrate binding site. Except for only one active site (8-15), whose sequence is RHTADYHP in Murcott and RPTANFRP in Fortune, all other active sites and binding sites are highly conserved between Murcott and Fortune.
